# Partially different? The importance of general equilibrium in health economic evaluations: An application to nocturia

**DOI:** 10.1002/hec.4638

**Published:** 2022-11-24

**Authors:** Marco Hafner, Erez Yerushalmi, Fredrik L. Andersson, Teodor Burtea

**Affiliations:** ^1^ RAND Europe Cambridge UK; ^2^ Birmingham City Business School Birmingham City University Birmingham UK; ^3^ International PharmaScience Center Ferring Pharmaceuticals A/S Copenhagen Denmark; ^4^ Ferring International Center SA Saint‐Prex Switzerland

**Keywords:** health economic evaluations, nocturia, partial general equilibrium, sleep, urology

## Abstract

Both the human capital approach and the friction cost approach are frequently used to quantify the productivity costs associated with illness, disability or death in health economic evaluations. In this paper we argue that these approaches have one major, but common shortcoming: they only capture partial equilibrium (PE) effects and therefore underestimate the true potential productivity costs associated with health conditions. They neglect the sizable, indirect, ripple effects in the economy captured by general equilibrium (GE) models. To demonstrate our point, we compare a traditional PE with a GE approach for the application to nocturia, a condition characterized by the need to frequently wake up at night to urinate. Nocturia is associated with substantial impairment of daytime functioning and work productivity. We employ large‐scale United Kingdom (UK) employer‐employee survey data to estimate the prevalence and productivity loss. These estimates are then used as shared inputs to drive both approaches. We find that the traditional PE approach underestimates the annual productivity cost of clinically relevant nocturia by around 16%. We propose a generalized GE/PE multiplier to approximate the GE effect for other health conditions. Our findings stress the importance of accounting for sizable GE effects when conducting health economic evaluations.

## 1 INTRODUCTION

Health economic evaluations which take a societal perspective require that all costs and benefits are considered, independent of who incurs them (e.g., the payers, patients). Failure to do so could lead to biased cost assessments (Edejer et al., 2003; Jönsson, 2009; Krol et al., 2013; Walker et al., 2019). Generally, costs can have different components, including direct healthcare costs, costs to patients and their caregivers, and productivity costs. Usually, productivity costs are measured through paid and unpaid production losses related to illness, disability or death. It has been shown that productivity losses contribute to large proportions of the overall costs associated with health conditions (Krol et al., 2016; Luengo‐Fernandez et al., 2013).

To measure the productivity costs associated with a health condition through lost productive time, two common approaches are applied: the human capital approach (HCA) and the friction cost approach (FCA), with a debate over which method is best suited to capture productivity costs (Kigozi et al., 2016, 2017; Koopmanschap et al., 1995; Koopmanschap & Rutten, 1996; Koopmanschap & van Ineveld, 1992; Targoutzidis, 2018). Both HCA and FCA can be applied in cost‐effectiveness, cost‐utility or cost‐of‐illness analyses that take a societal perspective (Drummond et al., 2015; Hubens et al., 2021; Pritchard et al., 2000).

In the HCA, the productivity costs associated with premature death are generally calculated as the loss of productivity estimated at the present value of future economic production over the expected remaining lifetime of an individual. The morbidity costs are calculated as the value of lost production due to acute illness or short‐ to long‐term disabilities. This can include lost production related to paid work but also to unpaid work, including for instance household production. The value assigned to lost production time related to paid work is usually gross earnings, which includes pay‐roll taxes. The rationale, according to economic theory, is that employers compare the marginal cost of worker time with the expected marginal productivity per worker. Time spent in unpaid work is usually valued in the HCA through an imputed wage based on the opportunity cost assumption (Hoefman et al., 2013; Pike & Grosse, 2018; Tranmer et al., 2005).

The FCA, on the other hand, limits the cost of lost production to the time required by the employer to adapt and resume production to levels without illness, disability, or death, that is, the so called “friction period”. The idea is that employees who leave the labor market because of illness, disability or death can be replaced by another employee (e.g., within or another firm) or an unemployed individual. From the employer's perspective, the productivity cost is the loss to find a replacement and train them (Koopmanschap et al., 1995; Koopmanschap & Rutten, 1996; Koopmanschap & van Ineveld, 1992).

These two common approaches have a major shortcoming: they only capture *partial equilibrium* (PE) effects. In a critical paper, Beutels et al. (2008) succinctly summarize our understanding of why PE models may underestimate the true costs of illness, disability or death. PE methods omit the potential spillover effects that health problems and diseases have on the wider economy, for example, on other individuals, other firms, the government, other markets and international trade links. The illness of individuals can adversely affect the productivity of other laborers and capital, disrupt supply chains and ripples across to other markets and across borders. Beutels et al. (2008) focus on large public health crises such as SARS to explain why PE underestimates these large health events. They therefore encourage the use of *general equilibrium* (GE) macroeconomic models, to capture these ripple effects—at a country and global level—and take into consideration that a population's health is not just an isolated factor for a country's healthcare sector but affects the whole economic system. Currently, the COVID‐19 pandemic is a global event whose cost cannot be accurately measured with PE and requires a GE approach.^1^ But beyond large‐scale public health events, research has shown the importance of considering GE effects for modeling the economic impacts associated with health conditions or health policy interventions (Hafner et al., 2017, 2020c; Kambou et al., 1992; Keogh‐Brown et al., 2010; Rutten & Reed, 2009; Smith et al., 2005; Taylor et al., 2014; Ye et al., 2006; Yerushalmi et al., 2019). However, to our knowledge, no study has yet directly assessed the different quantitative outcomes of a PE versus a GE approach for the same health condition.

The aim of our paper is, therefore, to compare the productivity costs of a health condition using a common PE approach versus a GE approach that uses the same underlying input data. Our purpose is to assess the discrepancy and raise awareness of the potential relative importance of GE multiplier effects in health economic evaluations. Currently GE studies in health economics are becoming more common, yet still not frequently utilized in health economic evaluations.

As an application, we focus on *nocturia*, a common but often overlooked condition. Nocturia is characterized as waking at night to pass urine at least once during the main sleep period (Hashim et al., 2019) and is regarded as one of the most bothersome lower urinary tract symptoms (Agarwal et al., 2014; Chapple et al., 2006; Kupelian et al., 2012).^2^ While there are many individual factors associated with inadequate sleep (e.g., bad sleep hygiene, chronic sleep disorders such as insomnia or sleep apnea), frequently having to wake up at night to urinate fragments sleep and disrupts the normal sleep cycle. Sleep fragmentation has adverse implications for the most restorative sleep stages and therefore is associated with negative consequences for an individual's health, wellbeing, daytime functioning and work productivity (Bliwise et al., 2015; Bonnet & Arand, 2003; Stepanski, 2002).

To compare PE with GE, using nocturia as an example, we apply an HCA to assess the productivity cost of illness. Our analysis consists of four analytical steps: First, we apply routinely used econometric methods to estimate the individual productivity loss associated with nocturia in the working population. Our employer‐employee dataset from the United Kingdom (UK) allows us to adjust for more confounding factors than previous econometric studies on nocturia. Next, we quantify the PE costs associated with nocturia, traditionally used in health economic evaluations, by multiplying a productivity loss parameter (e.g., measured in working time lost) with a measure of economic output such as gross domestic product (GDP) per capita. Third, we integrate the same parameter inputs applied for the PE approach into an economy‐wide GE model to make an equivalent comparison and report the differences between GE and PE cost estimates. We report these differences in the ratio between the GE versus PE cost estimates, which we term the GE/PE multiplier. Fourth, we simulate both GE and PE approaches using all combinations of productivity and prevalence values possible for health conditions in general, not just nocturia. We report a generic GE/PE multiplier grid that enables PE HCA calculations to be adjusted to approximate a GE HCA, which could lead to more accurate assessments of productivity costs in future health economic evaluations, without the need to develop a “full blown” computable GE model for each application.

For the *clinically relevant* nocturia example, which is defined as two or more nocturnal voids per night, we estimate its cost in terms of the UK's GDP at around £1.63 billion (bln) per year, when using a PE approach. But with a GE approach, this cost rises to £1.9 bln. This is a multiplier of 16% due to the indirect GE effects inherent in the economy. Furthermore, depending on the appropriate combination of disease‐specific marginal productivity cost and prevalence, we find the generic GE/PE multiplier grid to range between 3% to around 16%.

Finally, other literature has discussed alternative multipliers that account for the broader productivity impact. For example, a seminal paper by Pauly et al. (2002) introduces a wage multiplier that links an individual's cost of productivity with the overall team's productivity. Since individuals work complementary with others, and have differentiated jobs, skills, and variable ease of replacement by the manager, the productivity loss resulting from an individual could exceed those usually approximated by wages if team work is involved. Thus, individual's productivity could negatively affect the entire team's productivity and the economy overall. Nicholson et al. (2006), Pauly et al. (2008) and Zhang et al. (2017, 2015) empirically support this using various large employer‐employee surveys. In our paper, this type of feature is not explicitly modeled in the PE or GE models, though possible. We would expect the GE/PE multiplier to rise further because the productivity costs have non‐linear effects in the GE model and linear effects in the PE.

## 2 BACKGROUND

Nocturia is generally defined as waking at night to pass urine during the main sleep period. In line with other studies, and following general nocturia guidelines (Hashim et al., 2019) we focus on two relevant *nocturia* thresholds based on the observed number of nocturnal voids: (i) nocturia defined as one or more nocturnal voids; and (ii) nocturia defined as two or more nocturnal voids, where the non‐nocturia population is based on individuals reporting less than two voids per night. The latter definition is usually considered as clinically relevant nocturia.

Nocturia has different etiologies which are associated with distinct risk factors. For example, it can be caused by excessive fluid intake before bedtime. However, the most common factors are large urine volume produced during the night (nocturnal polyuria) or a reduced bladder capacity (Bliwise et al., 2019). At any given point in time, on average, up to 50% of the adult population report having to get up at night to urinate at least once, whereas up to 20% experience the need to get up at least twice (Bosch & Weiss, 2010).

Nocturia is commonly perceived as a problem of elderly men. Yet, while this condition does increase with age, it is relatively common among younger adults (Cornu et al., 2012; Wein et al., 2002). In fact, in younger years nocturia tends to be more prevalent among women, and in older age the prevalence is higher in men. Even among individuals aged 20–30, about 30% of women and 30% of men experience regularly the need to get up at night to urinate at least once (Bliwise et al., 2019).

Nocturia is associated with negative health consequences, such as increased risk of cardiovascular disease, depression and—in older individuals—a higher risk of injury through falls (Asplund, 2005; Chartier‐Kastler & Chapple, 2006; Weidlich et al., 2017). Due to the associated underlying chronic health conditions and the higher fall risk for the elderly, nocturia is also associated with higher relative mortality risk, but the causal pathway between nocturia and mortality remains unclear (Pesonen et al., 2020). The challenge with nocturia is that doctors and health practitioners often overlook nocturia as a potential health problem associated with sleep loss, and individuals that suffer from it often do not report it until it becomes unbearable, substantially affecting individual quality of life. Younger people with nocturia often do not feel comfortable talking about it, and many late‐middle age people just see nocturia as part of getting older (Marinkovic et al., 2004).

As the key complaint of nocturia is disrupted sleep, the consequences of nocturia are felt during the daytime, especially for the population that is active in the labor market. The sleep disruption associated with nocturia has been linked to daytime fatigue, decreased concentration, cognitive impairment, lower performance at work and higher levels of absenteeism and presenteeism (Asplund, 2005; Bliwise et al., 2019; Miller et al., 2016).

Aside from a few studies on nocturia that estimate the direct healthcare burden of nocturia, (e.g., Andersson et al., 2016; Tikkinen et al., 2010; Weidlich et al., 2017; Zeng et al., 2019), and predominantly focus on the elderly population (Dmochowski et al., 2019), the relationship between nocturia and productivity loss in the working population is an understudied issue. Furthermore, to our knowledge, there is very little empirical work on the macro‐level impact of nocturia beyond its effect on healthcare expenditure (see Holm‐Larsen, 2014; Jhaveri et al., 2019; Weidlich et al., 2017). These studies all use PE methods.

## 3 METHODOLOGY

In line with previous research and general nocturia guidelines, we consider two relevant nocturia definitions based on different nocturnal void thresholds $v=\left(1+,2+\right)$ whereby: (i) individuals with one or more nocturnal voids (*v* = 1+), are considered the population with nocturia and cost estimations are relative to a population with zero nocturnal voids; and (ii) individuals with two or more nocturnal voids (*v* = 2+), considered the *clinically relevant* nocturia population, where cost estimations are relative to a population with one or zero nocturnal voids.^3^

### 3.1 Econometric analysis to quantify the individual work productivity loss

#### 3.1.1 Data

We use UK employer‐employee data based on *Vitality UK's Britain's Healthiest Workplace (BHW) survey,* which has been collected annually since 2014 among UK companies and their employees.^4^ The BHW survey is administered among UK organizations with 20 or more employees and provided by organizations to their workforce.^5^ Employees are surveyed about over 100 questions related to socio‐demographic factors (e.g., age, gender, ethnicity, education, income, marital status, informal carer responsibilities), lifestyle behavior (e.g., nutrition, smoking habits, alcohol consumption, physical activity, sleep behavior), health factors (e.g., mental and physical health indicators, body mass index, chronic and musculoskeletal conditions), subjective well‐being and work productivity.^6^ Organizational participation in the survey is advertised in UK news outlets and through the network of Vitality's client base, an insurance and financial services company. Companies and their employees voluntarily participate and within companies, employee survey participation was encouraged through emails, newsletters and direct engagement with human resource managers.^7^

We use the BHW survey waves for the year 2017 and 2018 as they provided unique questions relating directly to nocturia, where survey participants were asked about the frequency of nocturnal voids. Our analysis uses a retrospective observational cross‐sectional study design, where data are pooled across the two waves. We only include workers aged 20–65 and exclude those on zero‐hours flexible work contracts. We also exclude female workers that report to be pregnant at the time of the survey response. The final sample based on the pooled cross‐sectional BHW data includes 52,887 observations across 285 unique companies, with 57 companies participating in both survey waves.^8^ Due to the nature of the cross‐sectional survey design and data collection process, the sample of firms participating in the survey is not fully representative for UK establishments. Moreover, it was not possible to longitudinally link employees within and across firms over the two survey waves. However, at the employee level, the BHW survey samples in general closely resemble other representative UK working population samples across a multitude of socio‐demographic and health characteristics (Stepanek et al., 2019). More detailed information about the data collection process can be found in BHW (2019) and applications using the data in Stepanek et al. (2019); Hafner et al. (2020a, 2020c).

#### 3.1.2 Empirical approach

We empirically estimate the association between nocturia, measured through the nocturnal void threshold definitions *v*, and productivity *w*, which can be illustrated as follows:

(1) $$w_{\mathrm{ict}}=\alpha_{v}v_{\mathrm{ict}}+\beta X_{\mathrm{ict}}+\delta_{c}+\gamma_{t}+\mu_{\mathrm{ict}}$$

where *w_ict_* is the observed productivity, measured as work impairment due to absenteeism and presenteeism,^9^ of individual *i* employed in company *c* at time *t*. Related to nocturia, the BHW survey data asks individuals about the number of nocturnal voids (“*How often do you usually get up during the night to go to the bathroom?*”) with the continuous variable based on applied standard scales from voiding diaries applied in nocturia patient‐reported outcomes research (Carney et al., 2012; Hsu et al., 2015; Romano et al., 2019). The maximum number of nocturnal voids reported among the sample population is five nocturnal voids.

As previously stated, we test for two different nocturia definitions$v=\left(1+,2+\right)$: (i) general nocturia, and (ii) clinically relevant nocturia. Our key parameter of interest is *α*_*v*_, the marginal change in work impairment (productivity) associated with nocturia among the sampled working population. Another parameter is *θ*_*v*_, the prevalence of nocturia among the (working) population.

The dependent variable *w*_*ict*_ is represented empirically as the sum of the percentage of working time missed due to sickness absence (absenteeism) and the percentage of work time adversely affected by productivity impairment while at work (presenteeism). This data was assessed with the Work Productivity and Activity Impairment‐General Health (WPAI‐GH) questionnaire. WPAI is a validated instrument to measure work productivity loss (Prasad et al., 2004; Reilly et al., 1993), and frequently applied to assess health‐related productivity loss measured through absenteeism and presenteeism (Brunner et al., 2019). The instrument consists of different questions with a recall time frame of 7 days. The questions asked the respondent about the total hours worked; the number of hours missed from work due to health reasons; the degree to which the respondent feels that a health problem has affected productivity while at work; and their ability to do daily activities other than work. Following the coding and scoring rules of the instrument, WPAI outcomes were expressed as impairment percentages, due to absenteeism and presenteeism, where higher percentages indicate greater impairment and lower productivity.

Furthermore in Equation (1), *X_ict_* represents a large set of potential confounding variables that are available in the BHW survey. Based on previous evidence, we included those that are expected to be associated with work productivity as well as nocturnal voiding (Tikkinen et al., 2009; Yoshimura, 2012).

First, a number of socio‐demographic covariates are considered, including age, gender, ethnicity, education, income, irregular working hours (e.g., night shifts), working hours per week, marital status, informal care responsibilities for a child and engagement in voluntary or charity activities.

Second, a number of health and lifestyle covariates including body mass index, smoking status (current smoker), physical inactivity (performing less than 150 min per week), excessive salt intake (adding regularly more than a pinch of salt to a meal), excessive alcohol consumption (drinking more than 14 alcohol units of 10 ml/8 mg per unit), the number co‐morbid clinically diagnosed health conditions (cancer, asthma, heart disease, kidney disease, diabetes, hypertension), as well as the musculoskeletal condition the individual suffers (e.g., back pain). Mental health was assessed through the Kessler Psychological Distress Scale which measures different emotional states based on a six‐item scale (Kessler et al., 2002). In line with previous research, a dichotomous variable was generated taking the value one if the overall Kessler score across all items is above 13, which is generally applied as the threshold of medium to severe psychological distress and anxiety (Kessler et al., 2003).

Finally, one challenge when estimating the effects of nocturia is the bidirectional relationship between sleep and nocturia (Ancoli‐Israel et al., 2011). Previous research suggests that nocturnal voiding predicts poor sleep quality (Araujo et al., 2014). However, individuals suffering from sleep‐onset insomnia may also just get out of bed at night and go to the bathroom, without nocturnal voiding being the main reason for their rise out of bed and subsequent sleep fragmentation. Disrupted sleep could also be caused by other factors such as sleep apnea, which contributes to sleep fragmentation, and has been associated with nocturia episodes and increased urine production (Miyazato et al., 2017).

Our data does not include a direct question about chronic sleep problems like insomnia or sleep apnea. However, the data includes a question based on the Pittsburgh Sleep Quality Index (Buysse et al., 1989) on a 5‐point scale whether the individual generally has problems falling asleep (“*During the last 7 days, did you have problems falling asleep; 0 not at all—4 very much*”). We use this variable as a proxy for sleep‐onset insomnia and also control for whether an individual on average sleeps less than 6 h or more than 9 h, taking into account short and long sleep duration. Furthermore, our large set of control variables include factors which are strongly correlated with sleep apnea, such as age, gender and obesity (Prasad et al., 2018) and hence at least indirectly we control for sleep apnea as a potential confounder as well.

Each econometric model specification includes company fixed‐effects, *δ*_*c*_, which take into account any potential confounders at the company‐level. Through the inclusion of company fixed‐effects we only exploit variation across employees within the same employer and therefore potential selection problems at the employer‐level or the influence of other workplace factors (e.g., work environment) are mitigated to some extent. We also include time fixed‐effects *γ*_*t*_ controlling for the week, month and year when the online survey response by the individual was submitted. Finally, *μ_ict_* denotes an idiosyncratic error term.

The associations between nocturia and work impairment are examined based on different model specifications by Ordinary Least Squares (OLS) and Fractional Logit (FL) regression, whereas the latter takes into account that the continuous dependent variable *w_ict_* is bounded between 0 and 1 (Papke & Wooldridge, 1996). Furthermore, as *w_ict_* is a non‐negative count variable containing a larger mass of zeros (about 60% in the study sample), motivated by previous studies using the same dependent variable (Brunner et al., 2019; Zhang & Sun, 2021), we apply sensitivity analyses by transforming the dependent variable into a count variable measuring the weekly hours of work lost due to absenteeism and presenteeism^10^ and use negative binomial regression (NBR) and zero‐inflated negative binomial (ZINB) regression models. We also use two different two‐part regression models, both use a logistic regression for the probability of the dependent variable being 0, but one using a generalized linear regression with zero‐truncated negative binomial distribution for the non‐zeros (TWONB) and the other a generalized linear regression with gamma distribution for the non‐zeros (TWOG). If the marginal effects of NBR, ZINB and the two‐part models are similar in magnitude, the parameter estimates are robust against the choice of estimation method.

Descriptive statistics of the key variables included in the analysis are provided in Tables A2 and A3 of the Appendix. For all analyses statistical significance was assessed at a significance level of 5% with standard errors (se) clustered at the company level. All statistical analyses were conducted in STATA 17.^11^

#### 3.1.3 Data limitations

While using the BHW survey enables an estimation of the productivity loss associated with nocturia at the employee level, with statistical adjustment for a large set of covariates, a number of limitations have to be highlighted: First, data from the survey are self‐reported. This creates potential for the under‐reporting or over‐reporting of factors, in line with social desirability. For instance, the reported prevalence of certain lifestyle habits—such as smoking or alcohol consumption—may be underestimated and that of physical activity may be overestimated. Second, as we define the nocturia population based on the self‐reported frequency of nocturnal bathroom visits, the main variable in the analysis may be subject to inaccuracies since it has not been clinically diagnosed by a health professional. Third, due to the lack of a fully representative sampling design and the inability to observe employees longitudinally, selection effects at the employer and employee level and reverse causality issues could bias the nocturia productivity loss and prevalence parameters. Therefore, when interpreting the empirical results on the association between nocturia and productivity, caution should be applied with regards to causality.

However, despite these limitations, the overall message of our paper will not change given that both PE and GE approaches (discussed below) use the same parameter inputs to drive their results.

### 3.2 The partial equilibrium (PE) human capital approach (HCA)

A PE HCA generally calculates productivity costs associated with ill‐health, disability or death as the expected time lost (e.g., in paid work) multiplied with a measure of income that proxies for an individual's productivity level, for example, the present value of average earnings plus fringe benefits (e.g., bonus payments) in an economy or the GDP per capita (Basu, 2016; Duevel et al., 2020; Garattini et al., 2000; Lofland et al., 2001; Luengo‐Fernandez et al., 2013; Murphy et al., 1998; Pearce et al., 2015; Wieser et al., 2011; Yuasa et al., 2021; Zimovetz et al., 2012).

For our analysis of nocturia, we only examine the productivity effects related to morbidity as the existing evidence on the link between nocturia and mortality is ambiguous and the associated deaths of nocturia most likely relate to individuals above retirement age. The PE productivity costs per worker associated with nocturia in monetary terms, depending on the definition of nocturia $v=\left(1+,2+\right)$, is calculated by multiplying *α*_*v*_ the marginal work impairment loss from Equation (1) (in percentage change) by a productivity level *W*:

(2) $$\xi_{v}=\alpha_{v}W$$

Finally, the total economic productivity cost of nocturia (in monetary terms) is calculated as

(3) $${\text{TOTAL}\;\text{COST}}_{v}=\xi_{v}P_{v}$$

with *P*_*v*_ the number of working age individuals in the UK's population with nocturia. *P*_*v*_ = *θ*_*v*_POP with POP the country's population weighted by *θ*_*v*_ the proportional prevalence of nocturia among the working population for the two nocturia definitions *v*.

### 3.3 The general equilibrium (GE) human capital approach (HCA)

Computable general equilibrium (CGE) models have already been used in health economics in the application to HIV/AIDS (Thurlow et al., 2009), Malaria (Yerushalmi et al., 2019), antimicrobial resistance (CCA, 2019; Smith et al., 2005; Taylor et al., 2014), pandemic influenza and non‐communicable disease (Keogh‐Brown et al., 2010; Smith et al., 2011) and various health policy assessments (Borger et al., 2008; Hsu et al., 2015; Rutten & Reed, 2009; Yerushalmi & Ziv, 2020) and Covid‐19 (Keogh‐Brown et al., 2020).

In this paper, we link the productivity costs of nocturia through changes to the effective‐labor supply. Our core model is a standard static multi‐country CGE models based on Yerushalmi et al. (2019) and Hafner et al. (2020c) and follows the structure by Lanz and Rutherford (2016). The model is programmed in the computer program GAMS^12^ using the MPSGE solver by Rutherford (1999). Our Supplementary Information S1 provides more detail on the model structure and parameters. Technical readers are referred to Lanz and Rutherford (2016) for the core model's equations and computer code. Below, we provide a general description of the model, but expand on how we link nocturia with labor productivity which is our added contribution.

#### 3.3.1 The core model

Our model has two regions: the UK and the Rest of the World (RoW). For each region, we solve multiple equations simultaneously. Households are endowed with capital and labor, which they offer to firms in exchange for income. On the demand side, given their budget constraint, households maximize a multi‐level constant elasticity of substitution (CES) utility function by demanding goods that are locally produced or from foreign imports. Similarly, the government in each country is another economic agent that receives income from collecting taxes, tariffs, and *net* funds from the household and other countries. Governments provide public goods and services by purchasing commodities locally and from abroad. Finally, an investment account also demands commodities in a similar way. On the supply side, in each country, perfectly competitive economic sectors maximize a profit function. Sectors supply goods and services by demanding labor, capital and intermediate inputs modeled as a multi‐level CES production function. Trade linkages between countries enable exports and imports of goods and services using an Armington framework (Armington, 1969).^13^

The core model is calibrated to the GTAP 10a database (Aguiar et al., 2019). GTAP 10a includes social accounting matrices of 141 countries—a double entry accounting system for incomes and expenditures. We focus our attention on the UK and aggregate all other countries into one region, that is, RoW. Furthermore, the GTAP 10a reference year is USD 2014 and we convert this to GBP 2021 by calculating the UK GDP and inflating it to match UK GDP 2021. As a test, we use the chained USD implicit price deflator^14^ and convert to GBP using Purchasing Power Parity.^15^ We find the discrepancy between these two methods to be only 1.3%, negligible. Finally, GTAP 10a includes 65 separate sectors. To simplify our results and analysis, but without risking affecting them, we aggregate sectors into four main groups (i.e., agriculture, industry, services and healthcare) that supply final and intermediate goods.

#### 3.3.2 The link between nocturia and the labor supply

Our model links the morbidity costs of nocturia as the value of lost production of labor. As mentioned, perfectly competitive economic sectors *j* ∈ *J* produce goods using a multi‐level, differentiable, constant return to scale production function $Y_{j}=f\left(K_{j},N_{jq},L_{j}\right)$. Each sector demands the following inputs: capital *K*_*j*_, intermediate inputs *N*_*jq*_ that are produced by sector *q* ∈ *J*, and effective‐labor *L*_*j*_. All markets must clear (i.e., demand equals supply). Specifically, the labor market clears with labor supply equal demand, *L* ^*s*^ = *∑*_*j*_ *L*_*j*_.

Omitting subscript *j* for simplicity, two elements determine the effective‐labor supply

(4) $$L^{s}=\bar{L}\cdot E$$

(i) $\bar{L}$ the physical supply of labor (e.g., number of employed workers), augmented by (ii) their productivity level *E* that depends on the health status of individuals.^16^ Yerushalmi et al. (2019) and Hafner et al. (2020c) use a similar GE HCA to study the link between malaria and physical activity, respectively.

An increase in effective‐labor supply is manifested through the removal of prolonged periods of sickness or levels of presenteeism that reduce the effective‐labor workforce. For the baseline analysis, which simulates the economy under current nocturia prevalence, we normalize the productivity levels by *E* = 1. In the *counterfactual* analysis, we determine the economy‐wide costs by altering the productivity levels to hypothetically remove the burden of nocturia. For both counterfactual analyses, depending on the nocturia definition (*v* = 1+, 2+), productivity rises by *e*_*v*_ which raises the effective‐labor supply (i.e., *E*_*v*_ = 1 + *e*_*v*_ and $L_{v}^{s}={\bar{L}}_{v}\cdot E_{v}$). Finally, we compare the difference between the baseline and the counterfactual analyses to obtain the productivity cost of nocturia. Similar to Equation (2), the parameter *e*_*v*_ is obtained by

(5) $$e_{v}=\alpha_{v}\theta_{v}$$

calculated using the parameter estimates for the marginal productivity loss and prevalence of nocturia among the working population (Equation 1).

A key difference between the GE and PE models is that the productivity level measure *W* (e.g., income) in Equation (2) is fixed in PE but adjusts endogenously in GE based on micro‐founded economic theory (i.e., wages adjust to changes in the supply/demand of goods and the labor market). In summary, the GE approach quantifies the productivity costs beyond its direct effect (of multiplying the number of patients by their production loss) as in PE. Here, the prevalence of a health condition and its adverse effect on productivity levels ripples throughout the economic system, reaching indirectly other sectors and households. It would even spillover onto economies abroad, through trade linkages, indirectly adding additional costs to all countries.

Finally, as there is uncertainty related to the parameter inputs, we further test our model assumptions for GE and PE by applying a Monte‐Carlo simulation to randomly, independently, generate a range for *θ*_*v*_ and *α*_*v*_. For each counterfactual analysis we execute the model 5000 times.

## 4 RESULTS

### 4.1 Prevalence of nocturia (*θ*_*v*_) and work productivity (*α*_*v*_)

Table 1 reports the prevalence of nocturia, by the number of nocturnal voids, by gender, for the sample population of workers aged 20–65. About 50.1% of the population report zero nocturnal voids, 40.8% report one nocturnal void, 6.8% two nocturnal voids and 2.3% three or more nocturnal voids. Women tend to experience more frequent nocturnal voids than men. These estimates are roughly in line with those estimated for other countries and studies (Bosch & Weiss, 2010; Cornu et al., 2012; Jhaveri et al., 2019; Tikkinen et al., 2009).

**TABLE 1:** Prevalence of nocturia by number of nocturnal voids

Voids	Total (%)	Women (%)	Men (%)
0	50.1	47.7	52.5
1	40.8	42.1	39.5
2	6.8	7.6	6.0
3+	2.3	2.7	2.0
Observations	52,887	26,406	26,481

*Note*: Table entries based on a BHW pooled cross‐sectional (CS) sample for the years 2017 and 2018. Chi‐square test suggests a statistically significant different distribution between male and female (*p* < 0.01). The maximum number of nocturnal voids reported in the data sample is five nocturnal voids. Due to the relative small number of individuals reporting four or five nocturnal voids we have grouped them into one category of three or more voids.

Next, depending on the definition of nocturia $v=\left(1+,2+\right)$, *θ*_*v*_ is calibrated in Equations (2) and (5) for PE and GE, respectively as follows: *θ*_1+_ is 49.9% which is the proportion of workers reporting to experience more than one nocturnal void. Similarly, *θ*_2+_ is 9.1% representing the proportion of workers experiencing two or more nocturnal voids.

Next, Table 2 reports the main regression results for estimating the association between nocturia and the work impairment percentage loss, that is, *α*_*v*_ in Equation (1), which calibrates productivity loss in Equations (2) and (5). For both OLS and FL, each of the three columns report the results for different model specifications, adding more covariates. Panel A reports the estimates for nocturia defined as 1+ voids (vs. 0 nocturnal voids), whereas Panel B reports estimates for nocturia defined as 2+ (vs. ≤1 nocturnal voids).

**TABLE 2:** Associations between nocturnal voids and work impairment due to absenteeism and presenteeism (parameter *α*_*v*_, % loss)

	(1)	(2)	(3)	(4)	(5)	(6)
Parameter *α*_*v*_ (% loss)	OLS‐1	OLS‐2	OLS‐3	FL‐1	FL‐2	FL‐3
A. 1+ nocturnal voids (vs. 0 nocturnal voids)						
1+ voids	0.03056	0.01981	0.01601	0.02915	0.01876	0.01534
se	(0.00208)**	(0.00183)**	(0.00183)**	(0.00192)**	(0.00171)**	(0.00170)**
95% CI: Low	0.02647	0.01621	0.01241	0.02538	0.01541	0.0121
95% CI: High	0.03465	0.02341	0.01960	0.03292	0.02211	0.01867
B. 2+ nocturnal voids (vs. ≤1 nocturnal voids)						
2+ voids	0.05099	0.02891	0.02186	0.04288	0.02368	0.01766
se	(0.00396)**	(0.00352)**	(0.00352)**	(0.00286)**	(0.00265)**	(0.00268)**
95% CI: Low	0.04319	0.02199	0.01492	0.03728	0.01848	0.01241
95% CI: High	0.05871	0.03583	0.02879	0.04848	0.02888	0.02290
Covariates						
Company FE	Yes	Yes	Yes	Yes	Yes	Yes
Time FE	Yes	Yes	Yes	Yes	Yes	Yes
Socio‐demographics	Yes	Yes	Yes	No	Yes	Yes
Lifestyle & health	No	Yes	Yes	No	Yes	Yes
Sleep	No	No	Yes	No	No	Yes
Observations	52,887	52,887	52,887	52,887	52,887	52,887

*Note*: **p < 0.01, *p < 0.05. Standard errors (se) clustered at the company‐level. 95% confidence intervals (CI) provided. Dependent variable is percentage of work impairment due to absenteeism and presenteeism (e.g., % of working time lost). Note that estimates need to be multiplied by 100 to receive effects in percentage points. Data sample based on BHW pooled cross‐sectional (CS) sample of the years 2017 and 2018. All models in columns 1–6 are adjusted for company‐ and time (week, month and year of given survey response) fixed effects (FE). Models in columns (1) and (4) are adjusted for age, gender, ethnicity, education, income, marital status, caring responsibilities (child), irregular working hours, working hours, the prevalence of financial concerns or whether the individual performs voluntary or charity work alongside normal working hours. Models in columns (2) and (5) are additionally adjusted for smoking status, excessive salt intake, body mass index, alcohol consumption, physical activity levels, mental health (Kessler score), chronic health conditions (cancer, heart disease, kidney disease, hypertension, diabetes) and musculoskeletal conditions (e.g., lower and upper back pain). Models in columns (3) and (6) are also adjusted for problems falling asleep, as well as short and long sleep duration.

Abbreviations: OLS, ordinary least squares; FL, fractional logit.

Depending on the estimation methods, the results show that adjusted for company and time fixed effects, as well as socio‐demographic characteristics, lifestyle factors and chronic health conditions, nocturia defined as 1+ nocturnal void is associated with an increase in work impairment between 1.98% points (OLS‐2; 95% CI 1.62–2.34) or 1.88% points (FL‐2; 95% CI 1.54–2.22). Nocturia defined as 2+ nocturnal voids is associated with an increase in work impairment between 2.89% points (OLS‐2; 95% CI 2.2–3.58) or 2.4% points (FL‐2; 95% CI 1.85–2.89). For example, a full‐time worker with 2+ nocturnal voids working 37.5 h loses on average about 0.9 h (54 min) of their total working time per week due to absenteeism and presenteeism, compared to workers with ≤1 voids per night.^17^

Adding a sleep‐onset insomnia control variable, and short and long sleep duration, reduces the point estimates for both definitions of nocturia further. For example, productivity for 1+ nocturnal voids falls to 1.6% points (OLS‐3; 95% CI 1.24–1.96) and to 1.53% points (FL‐3; 95% CI 1.2–1.87). For 2+ nocturnal voids, it falls to 2.19% points (OLS‐3; 95% CI 1.49–2.88) and 1.77% points (FL‐3; 95% CI 1.24–2.29). The reduction in magnitude between point estimates presented in OLS‐2/FL‐2 and OLS‐3/FL‐3 highlights the importance of taking into account potential sleep disorders as confounding factors when estimating the associations between nocturnal voiding and productivity loss. The UK employer‐employee survey data, with information on the prevalence of nocturnal voids, enables us to adjust for a larger number of relevant covariates that simultaneously may affect nocturnal voiding and work productivity. A limited number of similar studies find higher productivity losses of between 5% and 39% points for individuals with clinically relevant nocturia (Jhaveri et al., 2019; Kobelt et al., 2003; Miller et al., 2016; Weidlich et al., 2017). However, these studies do not adjust for as many covariates as we do, and hence likely overestimate the magnitude of the work impairment associated with nocturia. Our estimates, are therefore likely more conservative than estimates from previous studies.

We also perform robustness checks. For example, we test whether our results depend on the choice of estimation method. We transform the dependent variable into a count variable corresponding to the weekly hours lost due to absenteeism and presenteeism using NBR, ZINB and two‐part models. Results are shown in Table A4 of the Appendix. Column 1 and 2 replicate our baseline estimates using the transformed dependent variable using OLS (OLS‐2T and OLS‐3T). Based on the same covariate specifications and reporting the marginal effects at the mean characteristics of the covariates, columns 3 and 4 report the NBR, columns 5 and 6 report the ZINB, and columns 7–10 report the results for the two‐part models. The comparison shows that the models lead to similar marginal effects and hence suggesting that the results are not driven by the choice of the model.

### 4.2 Comparing the productivity costs of nocturia: PE versus GE

We compute and compare the productivity costs associated with nocturia between the PE and GE using the same set of inputs for both approaches. Table 3 summarizes our main parameter inputs for the two nocturia definitions: 1+ and 2+ nocturnal voids, with their upper/lower values used for the Monte‐Carlo sensitivity analysis. The productivity loss *α*_*v*_ is taken from FL‐3 in Table 2, which is the most conservative, with 1+ voids at 1.53% points (95% CI 1.21–1.87) and for 2+ voids at 1.77% points (95% CI 1.24–2.29).

**TABLE 3:** Main calibration parameters for partial equilibrium (PE) and general equilibrium (GE) with Monte‐Carlo simulation sample range

Parameter	Description		Mid value	Upper/lower values	Source
*α*_*v*_	Average productivity loss	1+ voids: 2+ voids:	1.534% 1.766%	(1.210%–1.867%) (1.241%–2.290%)*	Table 2
*θ*_*v*_	Prevalence per scenario	All 1+ to 0: All 2+ to 1 or 0:	49.9% 9.1%	(−25% to 25%)**	BHW survey; Table 1 and void threshold *v* = 1+, 2+
*W*	GDP per capita (2021)		£32,555		ONS
*P*_*v*_	Number of working age nocturia patients (millions)	1+ voids: 2+ voids:	15.6 mln 2.8 mln		Combining *θ*_*v*_ and employment stock (ONS).

*Note*: The table summarizes the calibrated inputs *θ*_*v*_ and *α*_*v*_ used in the PE and GE models. * 95% Confidence Interval (CI); ** +/− 25% upper and lower value.

In addition, to be able to compare the output of the GE and PE calculations, we compute the productivity cost of nocturia in terms of lost economic output measured through GDP. We therefore use the 2021 UK GDP per capita and set *W* to £32,555 (Office of National Statistics UK—ONS).^18^ Using the 2021 UK employment stock of 31.18 million (ONS) and nocturia prevalence *θ*_*v*_ (Table 1), we estimate that around 15.6 million (mln) adults in the UK workforce experience on average at least one nocturnal void, and about 2.8 mln experience two or more nocturnal voids.

We summarize the results for PE and GE in Table 4: Starting with the PE approach, the productivity cost per worker for nocturia defined as 1+ nocturnal voids is £499 (95% CI £391–£608) and for the clinically relevant nocturia threshold (2+ voids) at £575 (95% CI £404–£746). In other words, based on these productivity loss calculations, if a treatment for nocturia costs less than £575 per worker (e.g., including physician meetings, medicine and associated treatments), the net‐benefit for the economy would be positive. In terms of aggregated GDP, the sum of the productivity cost across the nocturia working population is £7.7 bln in annual GDP (95% CI £6.1 bln–£9.5 bln) for the 1+ void nocturia population and £1.6 bln (95% CI £1.2 bln–£2.1 bln) for the 2+ voids nocturia population.

**TABLE 4:** The productivity costs of nocturia, a comparison of PE and GE (GBP, 2021, https://www.ons.gov.uk/economy/grossdomesticproductgdp/timeseries/abmi/ukea)

	GDP loss (bln £)	% of GDP^a^	95% CI (bln £)	Cost per worker (£)	95% CI
Partial equilibrium (PE)					
1+ voids	7.7	0.35	(6.08–9.46)	499	(391–608)
2+ voids	1.63	0.07	(1.15–1.63)	575	(404–746)
General equilibrium (GE)					
1+ voids	8.9	0.4	(7.3–10.5)	571	(467–675)
2+ voids	1.9	0.09	(1.5–2.3)	665	(524–805)
GE/PE ratios					
1+ voids	1.14		(1.11–1.19)	1.14	(1.11–1.19)
2+ voids	1.16		(1.08–1.30)	1.16	(1.08–1.30)

*Note*: Voids 1+ assumes all workers with 1+ voids become 0 voids; Voids 2+ assumes all 2+ workers with nocturia become 0 or 1 voids. The corresponding 95% confidence intervals (CI) are reported in parentheses.

^a^ UK GDP 2021 £2198 bln (ONS).

**FIGURE 1:**
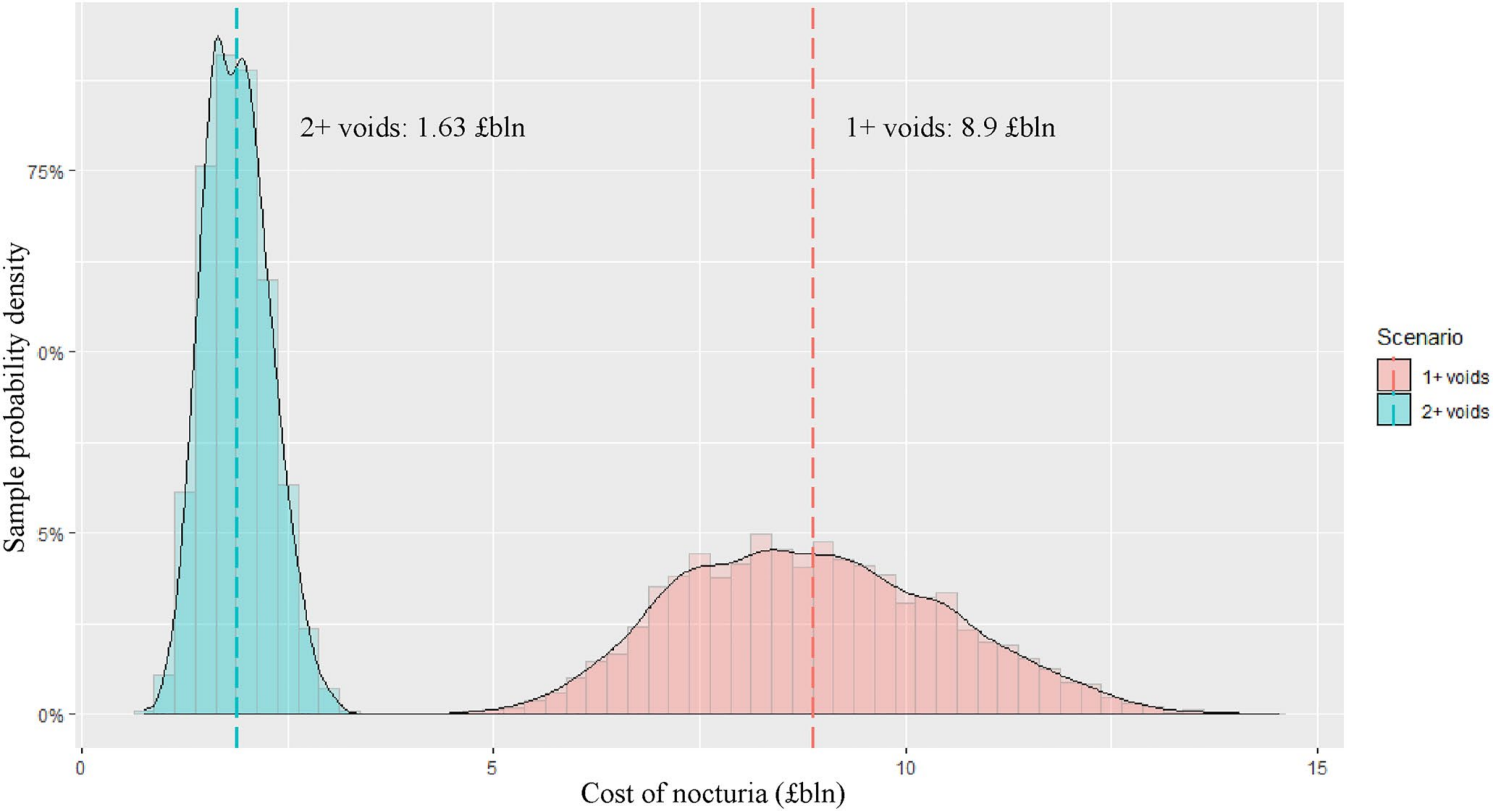
Lost economic value due to nocturia (PDF of the Monte Carlo simulation, £bln). The figure reports the Monte‐Carlo results of two counterfactual analyses: (i) the productivity costs associated with nocturia defined as 1+ voids; and (ii) clinically relevant nocturia (2+ voids), each executed 5000 times. The monetary cost of clinically relevant nocturia is around £1.63 bln.

Next, we compare these with the GE approach. We plot in Figure 1 the sample distributions for both nocturia definitions based on the Monte‐Carlo simulations. The total average cost to annual GDP is marked by the dashed lines. As expected, the distribution around 1+ voids is wider than for 2+ voids because it has one more element of variability (i.e., 1 void) and because 2+ voids is only 9.1% of the population.

For the 1+ voids nocturia population, the aggregated total cost in terms of lost GDP is £8.9 bln (95% CI £7.3 bln–£10.5 bln) annually, 0.4% of baseline GDP. We estimate the total cost in GDP terms for the clinically significant nocturia population (2+ voids) as £1.9 bln (95% CI £1.5 bln–£2.3 bln) or around 0.09% of baseline GDP. Per person, the productivity cost associated with nocturia defined as 1+ voids is £571 (95% CI £467–£675), and for nocturia defined as 2+ voids at around £665 (95% CI £524–£805).

Comparing the monetary estimates for the productivity costs using PE and GE with the same underlying input data, we find that the PE HCA underestimates the productivity cost associated with nocturia by between 14% and 16%. That is, in order to consider economy‐wide effects from the calculated productivity costs associated with a health condition, the traditional PE HCA would have to be multiplied by a GE/PE multiplier of 1.16 to approximate a GE HCA for the clinically relevant nocturia definition. Or in other words, health economic evaluations taking a societal perspective could considerably underestimate the true productivity costs leading to biased health economic evaluations.

### 4.3 A generalized GE/PE multiplier

The PE HCA is frequently applied to calculate the productivity costs associated with ill‐health or disability. It is an accessible and relatively straightforward approach that requires only a limited set of inputs related to a health condition (e.g., prevalence and marginal productivity loss). Yet, using a GE approach is crucial because it captures the additional ripple effects that improving population health has on the economy, which are omitted by PE. However, applying a “full blown” GE model is a less common skill and requires much higher computational demands.

Therefore, to *approximate* the full GE impact on the economy, when only using the PE HCA, we suggest utilizing a multiplier R following:

(6) GE=R⋅PE

Therefore, for any health conditions, we can estimate R by simulating the two models (discussed in Sections 3.2 and 3.3) with all possible combinations of *α* and *θ*, to obtain their HCA ratio by

(7) $$R=\frac{GE}{PE}=\frac{f\left(e\left(\alpha ,\theta \right),\Psi \right)}{g\left(\alpha ,\theta ,\Psi \right)}$$

with *Ψ* being the input data that both models are calibrated upon.

**FIGURE 2:**
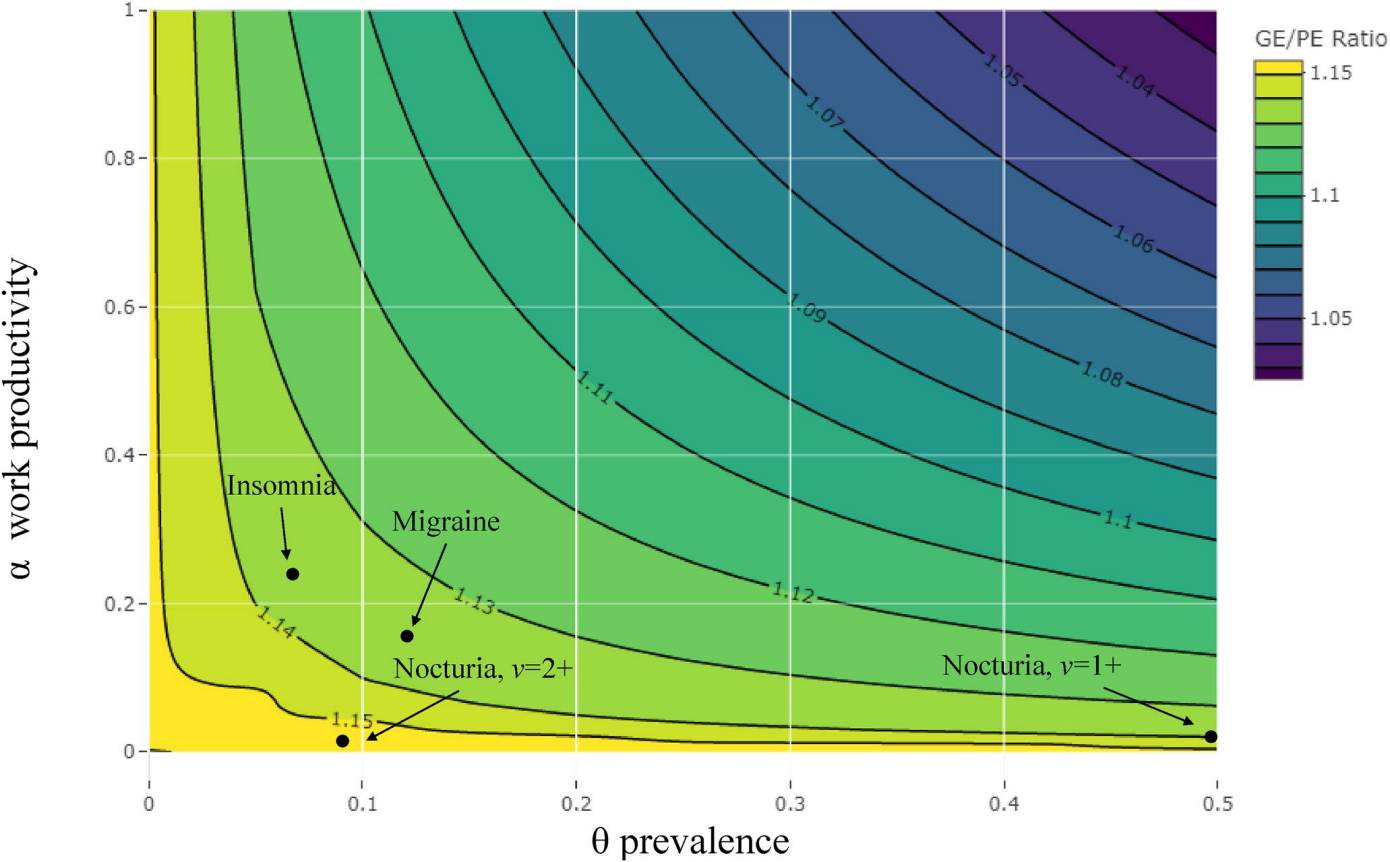
General equilibrium (GE)/partial equilibrium (PE) Ratio under different combinations of *α* and *θ*. The figure reports the contour plot of the GE/PE ratio R, for the two main parameters of the model, marginal productivity *α* and prevalence *θ*.

Below, we numerically do this. For each model, we record their HCA, compute the ratio $R=h\left(\alpha ,\theta \right)$, and graph it on a contour plot. As discussed for nocturia, Figure 2 already records the two R ratios computed for *v* = (1+, 2+) at 1.14–1.16, respectively. For illustration, we consider two other health conditions, chronic insomnia and migraine. For chronic insomnia, in the UK, *θ* is around 6.8% (Perlis et al., 2020) with an associated *α* estimated at 23.8% of total working time lost due to absenteeism and presenteeism (DiBonaventura et al., 2015). For migraine, in the UK, *θ* is around 12% with an associated *α* of 15.4% of working time lost due to absenteeism and presenteeism (Vo et al., 2018). This would mean that to obtain the full GE HCA related to morbidity of chronic insomnia and migraine, the PE HCA should be multiplied by $R_{\text{insomnia}}=1.138$ and $R_{\text{migraine}}=1.135$, respectively.

Finally, in Figure 2, R falls as illness severity rises to the upper‐right (i.e., severity in terms of being more debilitating or higher prevalence). In this static GE model, capital supply is fixed and as the baseline illness severity rises, the gains in marginal productivity (of removing an illness) diminish and the capital‐to‐labor ratio falls. However, in the PE model, the capital‐to‐labor ratio is implicitly assumed constant, holding marginal productivity constant. In other words, changes in the PE model are linear, but non‐linear with GE. As long as the values for *α* and *θ* are relatively low (lower‐left quadrant), which is a realistic setup for many health conditions, the GE and PE approach simulate sufficiently well. However, in severe cases, both models would need to be updated, especially if they would need to consider the long‐run effects that health has on the economy.^19^

## 5 DISCUSSION

Our analysis demonstrates the importance of considering GE effects in health economic evaluations that take a societal perspective. While we highlight limitations to the estimated input parameters (e.g., non‐fully representative sample population, challenges establishing causality between nocturia and work impairment) the key finding of this study would not change given that both PE and GE use the same inputs to drive their results. We acknowledge that GE models are computationally more advanced and may require more resources, therefore we have provided an approximate GE/PE multiplier based on a set of given parameter inputs related to a health condition that can be utilized to adjust the productivity costs associated with morbidity based on a PE HCA.

In what follows, we discuss limitations and areas for future research. First, as discussed, our analysis of nocturia only focuses on the morbidity aspect of productivity costs because the evidence regarding mortality is ambiguous and perhaps not strongly linked to nocturia for a population that is active in the labor market. In addition, nocturia could also affect the physical supply of labor through early retirement decisions (Jhaveri et al., 2019). The BHW survey we use only covers individuals currently employed, and therefore we are unable to consider this potential effect. Considering these additional factors would likely lead to a larger estimated economic burden than we report. In general, for other health applications, mortality related productivity costs could be added into the PE and GE models through *P*_*v*_ in Equation (3) and ${\bar{L}}_{v}$ in Equation (4), respectively.

Second, we only focus on workers currently employed. However, individuals could be out of the labor force altogether because of the condition, or are currently permanent or temporary carers of individuals with the condition. We have not focused on the potential productivity costs of unpaid work in terms of carers because the evidence related to nocturia is scarce. However, the value of unpaid work can easily be included in a GE model. For instance, if carers are active in the labor market and lose days off work because of the carer duties, this could also be captured by *e*_*v*_ in Equation (5). Yerushalmi et al. (2019) use this approach to cost the days lost by parents/carers of children with malaria. Neglecting the effects of disease‐specific unemployment or the associated carer burden underreports the true costs.

Third, our GE analysis is focused on the HCA, a predominant method in health economic evaluations to assess productivity costs. However, the FCA could also be implemented in the GE model, for example, by introducing a pool of unemployed workers which could replace workers that fall out of the labor force, and include search costs or other costs related to hiring and firing employees within the suite of model equations that represent the decision of firms to hire workers.

Fourth, currently our GE analysis does not consider additional multiplier effects linking an individual's productivity with a team's productivity (Pauly et al., 2002). However, the GE model could be extended, for example, by incorporating a micro‐founded team‐specific productivity element that captures external economies within the firm's production function. We would expect the GE/PE multiplier to increase because the productivity costs have non‐linear effects in the GE model and linear effects in the PE.

Finally, to keep the comparison between PE and GE effects simple, we ignored the direct healthcare costs associated with nocturia and other illnesses. However, a GE can model this as expenditure on healthcare goods, either by public or private financing, which itself drives further demand effects. This is important as healthcare is not an isolated sector in the economy and demands intermediate inputs from many other sectors. Similarly, public financing could also create distortionary tax effects that need to be considered. Yerushalmi and Ziv (2020) use a GE model that differentiates public from private financing and production to assess these types of issues.

## 6 CONCLUSION

The main findings of this study highlight the magnitude of GE effects when assessing the potential productivity costs associated with health conditions. Failure to take into account these GE effects in health economic evaluations could lead to an underestimation of the costs associated with ill‐health and hence to biased assessments. Beyond productivity costs, if the societal perspective is important for economic evaluations, then the application of GE models which considers the interactions of different agents within an economic system can substantially improve our understanding of the wider economic impacts of ill‐health, direct healthcare spending or time spent in providing unpaid care. Future studies should therefore consider taking a GE approach instead of the PE approach prevalent in many health economic applications, or at least incorporate the range of GE/PE multiplier that we provide.

## CONFLICT OF INTEREST

Author Marco Hafner conducted this work as employee of RAND Europe, which received the funding for this research. Erez Yerushalmi did not receive funding for this work. Fredrik L. Andersson and Teodor Burtea are employees of Ferring Pharmaceuticals. The funding agreement ensured the authors' full independence in designing the study, interpreting the data, and writing the manuscript.

## Supporting information

Supporting Information S1

## Data Availability

The BHW data that support the findings of this study are available from Vitality UK. Restrictions apply to the availability of these data, which were used under license for this study. Data are available from the authors with the permission of Vitality UK.

## ENDNOTES

^1^
For example, Hafner et al. (2020b) use a multi‐country general equilibrium model to quantify the cost of COVID‐19 vaccine nationalism.

^2^
Note that individuals reporting less than two nocturnal voids are generally not regarded as having clinically significant nocturia (Bosch & Weiss, 2010; Tikkinen et al., 2010).

^3^
Note that the populations considered as not having nocturia differ across the two definitions.

^4^
See BHW (2019) for further details.

^5^
Its main purpose is to support employees in better understanding their health and well‐being via a personalized individual report whilst providing a comprehensive aggregated report to organizations to support their well‐being strategies and improvement for productivity.

^6^
All survey participants provided electronic written consent for anonymized usage of their records for research purposes as required. The ethics committee at the data collecting organization RAND Europe has reviewed and approved the data collection and research plan.

^7^
The average employee response rate to the survey within companies is about 33% in each wave.

^8^
A distribution of participating companies with additional company size information for both survey waves is provided in Table A1 of the Appendix.

^9^
With higher levels of work impairment reflecting lower productivity.

^10^
Calculated as the percentage of working time lost due to absenteeism and presenteeism times the reported weekly working hours.

^11^

https://www.stata.com/

^12^

www.gams.com

^13^
Armington is commonly used in CGE modeling and allows cross‐hauling of the same goods.

^14^

https://fred.stlouisfed.org/series/GDPDEF

^15^

https://data.oecd.org/conversion/purchasing-power-parities-ppp.htm#indicator-chart

^16^
Here, physical and efficiency are perfect substitutes in production, and only their combination $\bar{L}\cdot E$ matters for output. That is, the fixed number of physical persons $\bar{L}$ will not be a source of diminishing returns (Barro & Sala‐i Martin, 2003; Romer, 2000).

^17^
0.9 = 2.4/100 × 37.5 h/week

^18^
The UK 2021 GDP (Chained volume measures: seasonally adjusted) was £2198 bln, with population at 67,530,759 (ONS).

^19^
For example, if severity would be extremely high (e.g., *α* > 65% and *θ* > 90%), we would want to add additional components to the GE model to appropriately simulate demographic changes, dynamic capital investment, and labor‐leisure effects. In any case, for such severity levels, where the burden is affecting such a large proportion of the population, the PE model would poorly represent the link between health and the economy and a GE model is much better suited for quantifying the cost associated with ill‐health.

## Appendix

**TABLE A1:** Number of organizations in the BHW workforce survey

	2017	2018
Number of organizations	197	145
Share by organizational size (%)		
Small (<50 employees)	34.36	33.81
Medium (50–250 employees)	26.38	26.62
Large (250 + employees)	39.26	39.57
Average number of responses per organization	162	193

*Note*: The table provides the total number of participating organizations (employers) for the 2017 and 2018 BHW survey waves. Between 2017 and 2018, 57 organizations participated in both surveys with a total of 285 unique participating organizations. The mean number of employee responses across all organization are reported by wave.

**TABLE A2:** Variables from the BHW UK workforce survey

Variable	Description	Mean	SD	Min	Max
Work impairment					
Work impairment total	% Work time lost due to absenteeism and presenteeism	0.124	0.201	0	1
Work impairment absenteeism	% Work time lost due to absenteeism	0.008	0.055	0	1
Work impairment presenteeism	% Work time lost due to presenteeism	0.115	0.186	0	1
Work impairment total (zero)	Work time lost due to absenteeism and presenteeism = 0 (1 yes—0 no)	0.593	0.491	0	1
Work impairment absenteeism (zero)	Work time lost due to absenteeism = 0 (1 yes—0 no)	0.948	0.221	0	1
Work impairment presenteeism (zero)	Work time lost due to presenteeism = 0 (1 yes—0 no)	0.601	0.489	0	1
Socio‐demographics					
Female	Gender: Female (1 yes—0 no)	0.502	0.500	0	1
Age	Age (continuous)	39	11	20	65
White	Ethnicity: White (1 yes—0 no)	0.912	0.283	0	1
Asian	Ethnicity: Asian (1 yes—0 no)	0.014	0.116	0	1
Black	Ethnicity: Black (1 yes—0 no)	0.042	0.201	0	1
No tertiary education	Education: Less than university degree (1 yes—0 no)	0.399	0.490	0	1
Income (£, 1000s)	Annual income 1000£(continuous)	43.815	27.801	15	150
Divorced	Marital status: Divorced (1 yes—0 no)	0.036	0.187	0	1
Widowed	Marital status: Widowed (1 yes—0 no)	0.004	0.061	0	1
Child	Caring responsibility: Child younger than 18 (1 yes—0 no)	0.271	0.445	0	1
Irregular hours	Irregular working hours (e.g., night shifts (1 yes—0 no))	0.138	0.345	0	1
Working hours	Hours employee works in a typical work week	35.469	7.697	25	60
Financial concerns	Having financial concerns (1 yes—0 no)	0.081	0.273	0	1
Engaged	Engaged in voluntary activities or charity work outside work (1 yes—0 no)	0.258	0.438	0	1

*Note*: The table provides descriptive statistics of the pooled BHW survey waves 2017 and 2018 based on 52,887 observations. Provided are the mean, standard deviation (SD) and the minimum and maximum values for each variable.

**TABLE A3:** Variables from the BHW UK workforce survey (*continued from previous table*)

Variable	Description	Mean	SD	Min	Max
Lifestyle & health					
Smoker	Currently smoking (1 yes—0 no)	0.097	0.296	0	1
Excessive alcohol	Consumption >14 units (8 mg) per week (1 yes—0 no)	0.296	0.457	0	1
Physically inactive	Less than 150 min of activity per week (1 yes—0 no)	0.339	0.473	0	1
Excessive salt intake	Salt addition to every meal regularly more than a pinch of salt (1 yes—0 no)	0.043	0.203	0	1
Obese	Body mass index >30 (1 yes—0 no)	0.184	0.388	0	1
Overweight	Body mass index >25–30 (1 yes—0 no)	0.343	0.475	0	1
Underweight	Body mass index <18.5; (1 yes—0 no)	0.015	0.120	0	1
At risk of mental health problems	Kessler Psychological Distress scale >13 (1 yes—0 no)	0.064	0.244	0	1
MSK: Neck	Problem with neck during the last 12 months (1 yes—0 no)	0.329	0.469	0	1
MSK: Shoulder	Problem with shoulder during last 12 months (1 yes—0 no)	0.325	0.468	0	1
MSK: Elbow	Problem with elbow during last 12 months (1 yes—0 no)	0.068	0.253	0	1
MSK: Wrist/hand	Problem with wrist/hand during the last 12 months (1 yes—0 no)	0.164	0.369	0	1
MSK: Upper back	Problem with upper back during the last 12 months (1 yes—0 no)	0.152	0.359	0	1
MSK: Lower back	Problem with lower back during the last 12 months (1 yes—0 no)	0.444	0.497	0	1
MSK: Hip/thigh	Problem with hip/thigh during the last 12 months (1 yes—0 no)	0.146	0.354	0	1
MSK: Knee	Problem with knee during the last 12 months (1 yes—0 no)	0.280	0.449	0	1
MSK: Ankle/foot	Problem with ankle/foot during the last 12 months (1 yes—0 no)	0.185	0.388	0	1
Asthma	Clinically diagnosed with asthma within the last 12 months; (1 yes—0 no)	0.082	0.274	0	1
Heart	Clinically diagnosed with cardiovascular disease within the last 12 months; (1 yes—0 no)	0.012	0.110	0	1
Kidney	Clinically diagnosed with kidney disease within the last 12 months; (1 yes—0 no)	0.006	0.080	0	1
Cancer	Clinically diagnosed with cancer within the last 12 months; (1 yes—0 no)	0.005	0.069	0	1
Diabetes	Clinically diagnosed with Diabetes within the last 12 months; (1 yes—0 no)	0.017	0.129	0	1
Hypertension	Clinically diagnosed with hypertension within the last 12 months; (1 yes—0 no)	0.051	0.219	0	1
Sleep					
Insomnia	Difficulties falling asleep: (0 never—4 often)	0.918	1.149	0	4
Short sleep	Hours of sleep <6 (1 yes—0 no)	0.066	0.249	0	1
Long sleep	Hours of sleep >9 (1 yes—0 no)	0.022	0.148	0	1

*Note*: The table provides descriptive statistics of the pooled BHW survey waves 2017 and 2018 based on 52,887 observations. Provided are the mean, standard deviation (SD) and the minimum and maximum values for each variable.

Abbreviation: MSK, musculoskeletal.

**TABLE A4:** Robustness checks—Associations between nocturia and working hours lost due to absenteeism and presenteeism

	(1)	(2)	(3)	(4)	(5)	(6)	(7)	(8)	(9)	(10)
	OLS‐2T	OLS‐3T	NBR‐2	NBR‐3	ZINB‐2	ZINB‐3	TWONB‐2	TWONB‐3	TWOG‐2	TWOG‐3
A. 1+ nocturnal voids (vs. 0 nocturnal voids)										
1+ voids	0.685	0.548	0.714	0.572	0.730	0.674	0.734	0.593	0.735	0.594
se	(0.068)**	(0.068)**	(0.067)**	(0.067)**	(0.066)**	(0.066)**	(0.063)**	(0.063)**	(0.063)**	(0.063)**
95% CI: Low	0.552	0.414	0.584	0.440	0.601	0.544	0.611	0.469	0.611	0.469
95% CI: High	0.819	0.681	0.845	0.703	0.859	0.804	0.858	0.717	0.859	0.718
B. 2+ nocturnal voids (vs. ≤1 nocturnal voids)										
2+ voids	0.972	0.716	0.880	0.637	0.881	0.789	0.888	0.635	0.890	0.636
se	(0.124)**	(0.123)**	(0.094)**	(0.095)**	(0.099)**	(0.098)**	(0.097)**	(0.098)**	(0.097)**	(0.098)**
95% CI: Low	0.728	0.473	0.695	0.451	0.687	0.596	0.698	0.443	0.699	0.444
95% CI: High	1.215	0.959	1.064	0.823	1.076	0.982	1.078	0.827	1.08	0.829
Covariates										
Company FE	Yes	Yes	Yes	Yes	Yes	Yes	Yes	Yes	Yes	Yes
Time FE	Yes	Yes	Yes	Yes	Yes	Yes	Yes	Yes	Yes	Yes
Sociodemographic	Yes	Yes	Yes	Yes	Yes	Yes	Yes	Yes	Yes	Yes
Lifestyle & health	Yes	Yes	Yes	Yes	Yes	Yes	Yes	Yes	Yes	Yes
Sleep	No	Yes	No	Yes	No	Yes	No	Yes	No	Yes
Observations	52,887	52,887	52,887	52,887	52,887	52,887	52,887	52,887	52,887	52,887

*Note*: ***p* < 0.01, **p* < 0.05. Standard errors (se) clustered at the company‐level. Dependent variable is the number of working hours lost per week due to absenteeism and presenteeism (calculated as working time lost in % times working hours per week as reported by the employee). Data sample based on BHW pooled cross‐sectional (CS) sample of the years 2017 and 2018. All models in columns 1–10 are adjusted for company‐ and time (FE) fixed effects (week, month and year of given survey response). Model ordinary least squares (OLS)‐2T and OLS‐3T represent same models as reported in Table 2 columns 2 and 3 but with the transformed dependent variable.
