# Supplementary material for: Partially different? The importance of general equilibrium in health economic evaluations: An application to nocturia
Source: Health Econ. 2022 Nov 24;32(3):654–74. doi: 10.1002/hec.4638 (PMC10100343; doi:10.1002/hec.4638)
Supplement: Supplementary file 1 — Supporting Information S1 [file HEC-32-654-s001.pdf]

Online Supplementary Appendix:

1 The general equilibrium human capital approach: description of the multi-regional CGE model for nocturia

Our core model is a standard static multi-country CGE models based on Yerushalmi et al. (2019); Hafner et al. (2020b) and Hafner et al. (2020d). The model is programmed in the computer program GAMS[1] using the MPSGE solver by Rutherford (1999). The core model follows closely with Lanz and Rutherford (2016) that document the full set of equations and the corresponding computer code. Below we provide an overview description of the model, and encourage interested readers to look at Lanz and Rutherford (2016) for more detail.

The core model is calibrated to the GTAP 10a database (Aguiar et al., 2019). GTAP 10a includes social accounting matrices (SAMs) of 141 countries - a double entry accounting system for incomes and expenditures and 65 commodities. The data characterizes bilateral trade flows, intermediate demands, and tariffs/subidies on exports, imports and other indirect taxes and subsidies.

In the calibration, we focus on the United Kingdom and aggregate all other countries into the Rest of the World, $UK, ROW \in r$, respectively. Furthermore, because we are quantifying the cost of nocturia at a country-level, it is not necessary to complicate the model with many disaggregated levels of sectors. We therefore aggregate the 65 sectors in GTAP into four main sectors $j =$ [Agriculture, Industry, Services, Health] - with q alias to j.

Finally, the GTAP 10a reference year is USD 2014 which we convert to GBP 2021 by calculating the UK GDP and inflating it to match UK GDP 2021. As a test, we use the chained USD implicit price deflator[2] and convert to GBP using Purchasing Power Parity (PPP)[3]. We find the discrepancy between these two methods to be only 1.3% - negligible. Finally, GTAP 10a includes 65 separate sectors.

1.1 Model overview

The model solves multiple equations simultaneously and decisions about the allocation of resources are modeled by the canonical micro-economic optimization framework: (*i*) consumers maximize welfare subject to a budget constraint with fixed levels of investment and public expenditure; (*ii*) producers combine intermediate inputs and primary factors; and (*iii*) markets clear. Figure 1, which we adopt from Lanz and Rutherford (2016), provides an overview of the model structure and its calibration to the GTAP 10a database of social accounting matrices (SAMs) (Aguiar et al., 2019). It shows the circular flows within a single region as follows:

Starting at the top-left of Figure 1, firms produce goods in product markets. Moving clockwise, some of these goods are demanded by the firms themselves as intermediate inputs, while most are demanded as final consumption (private, investment, government) or exported abroad. Together, final demands make up the welfare of a representative agent RA. In each country, the representative agent is endowed with labor and capital which it provides to firms in exchange for income.

[1] www.gams.com
[2] https://fred.stlouisfed.org/series/GDPDEF
[3] https://data.oecd.org/conversion/purchasing-power-parities-ppp.htm#indicator-chart

Trade linkages between countries enable exports and imports of goods and services using an Armington framework, which is commonly used in CGE modeling to allow for the cross-hauling of the same goods (Armington, 1969). As Lanz and Rutherford (2016) explain in more detail, imports from different countries include transportation services, which enter on a proportional basis, to reflect differences in unit transportation margins across different goods and trading partners. The Armington composite therefore involves trading both imported goods and associated transportation services.

Finally, as Figure 1 illustrates in red font, we capture the effect of nocturia as a change in the effective labor-supply - discussed further in subsection 1.4.

Figure 1: Model structure overview per region

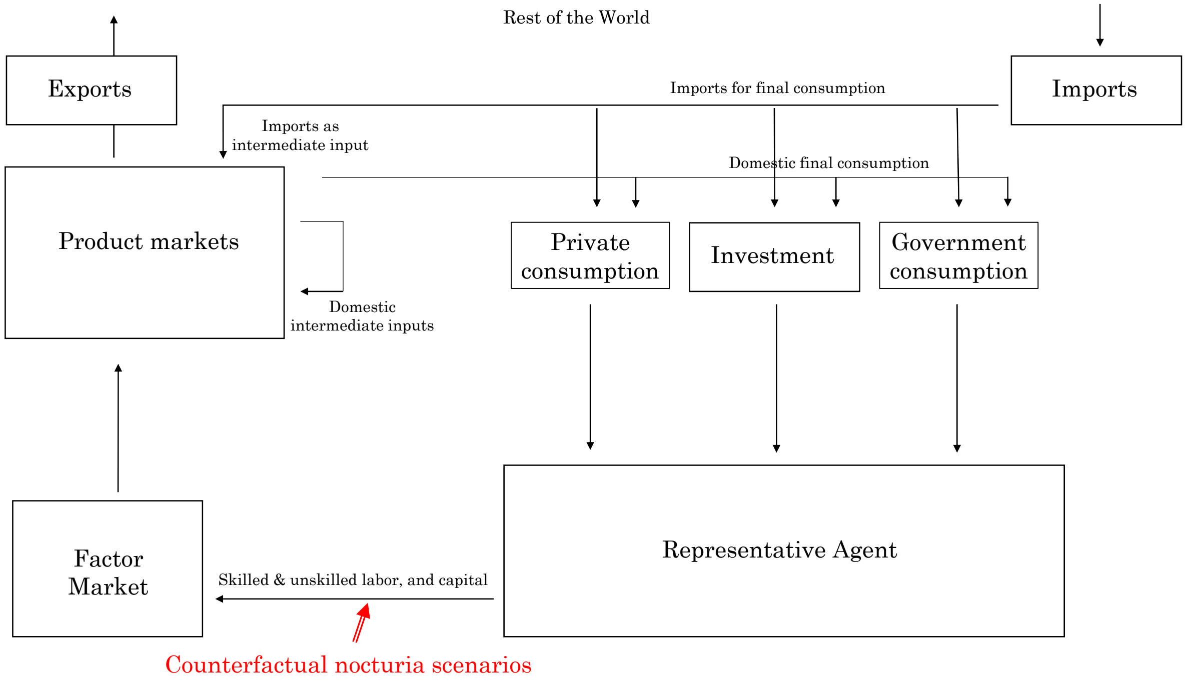

The figure provides an overview of the model structure. Demands are shown by arrow heads.

1.2 Goods supply

Omitting country index r, Figure 2 provides more detail on the firms' production function in the product market. The four main production sectors, discussed previously, are perfectly competitive economic sectors that produce goods using a multi-level, differentiable, constant return to scale (CRS) production function $Y_j = f(K_j, N_{jq}, L_j)$. Each sector demands the following inputs: capital K_j, effective-labor L_j, and intermediate inputs N_{jq} that are produced by sector q.

On the left-hand of Figure 2, firms demand input factors: skilled and unskilled labor and capital which they obtain from the factor market in Figure 1. At the lowest level, we aggregate skilled and unskilled labor with an inelastic substitution elasticity $\sigma = 0.5$ that characterizes their differences in skills. In the next level, aggregate-labor L_j and capital K_j are aggregated into a value added using a Cobb-Douglas function, as usually applied in many macroeconomic models.

The right-hand side of Figure 2 illustrates the demand for intermediate inputs in the production function. At the lowest-level, domestic goods and competing imports are aggregated to form an aggre-

Figure 2: Model structure overview per region

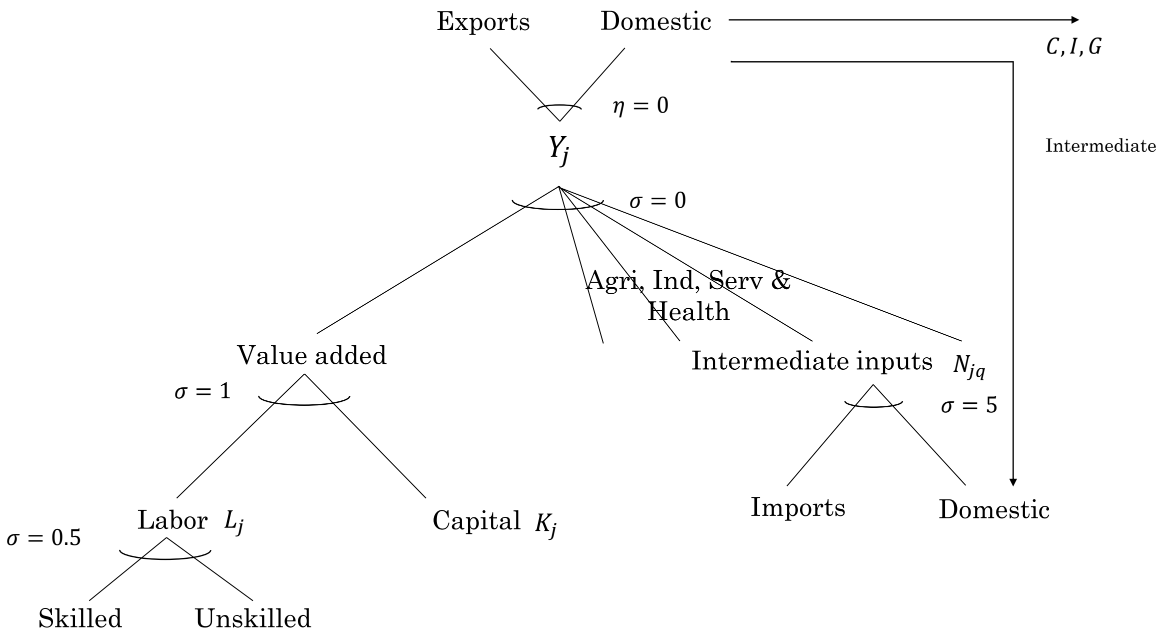

The figure illustrates the multi-level production function.

gate intermediate input of production. We use a high elasticity of substitution, $\sigma = 5$ to characterize the high degree of sustainability between these intermediate inputs. Next, at the top nest, intermediate goods and the value added are aggregated in fixed-proportions (i.e., a Leontief function) to form products. Finally, each product is consumed domestically by private consumption, investment, government consumption, or as intermediate good, or exported abroad.

1.3 Domestic demand

In each country, the representative agent RA_r is endowed with capital and labor, which they provide to firms in exchange for income, and also collect (provide) taxes (subsidies) on domestic goods, and tariffs on imports and export. With this income, they maximizes a multi-level CRS function, which we illustrate in Figure 3. On the left-hand side, RA_r demands private consumption with a two-level function. At the lowest-level, for various sectors, households buy domestic final goods or imports with a high substitution elasticity $\sigma = 3$. In the next level, goods are aggregated using a Cobb-Douglas function and provide an index of private consumption.

Similarly, but only partially illustrated in Figure 3, investment and government consumption also demand domestic final goods and imports. Finally, all types of consumption are aggregated in fixed proportions to form RA_r's welfare index.

Figure 3: Model structure overview per region

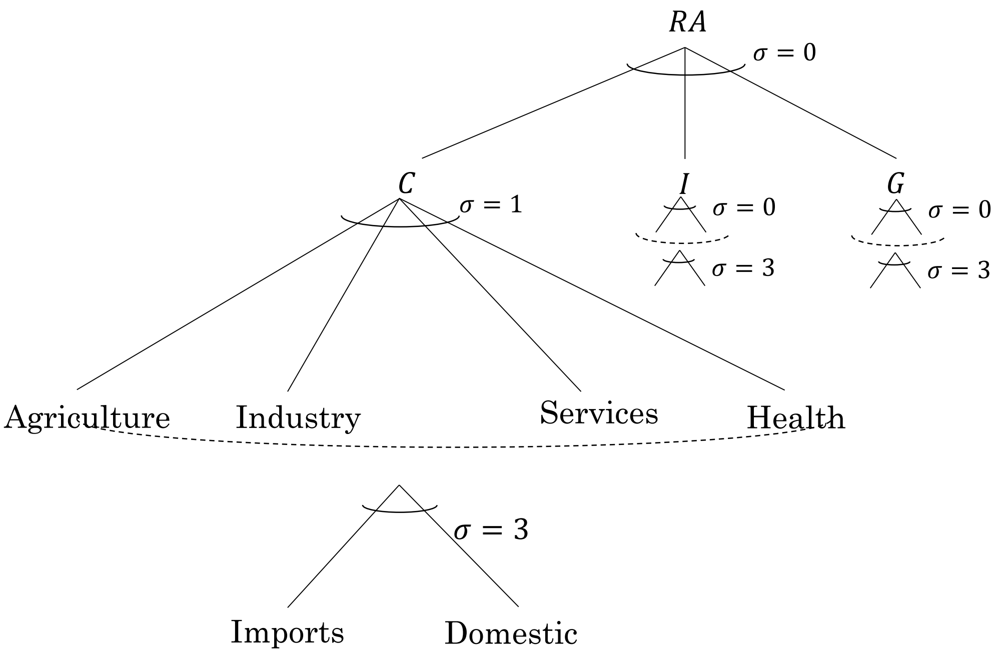

The figure illustrates the private utility function .

1.4 The link between nocturia and the labor supply

Our model links the morbidity costs of nocturia as the value of lost production of labor. Two elements determine the effective-labor supply

$$L^s = \bar{L} \cdot E \tag{1}$$

where (*i*) $\overline{L}$ the physical supply of labor (e.g. number of employed workers), augmented by (*ii*) their productivity level E that depends on the health status of individuals. In this format, physical and efficiency are perfect substitutes in production, and only their combination $\bar{L} \cdot E$ matters for output, i.e., the fixed number of physical bodies $\bar{L}$ will not be a source of diminishing returns (Barro and Sala-i Martin, 2003; Romer, 2000). Yerushalmi et al. (2019) and Hafner et al. (2020d) use a similar CGE human capital approach to study the link between malaria and physical activities, respectively.

In this general equilibrium model, all markets clear (i.e., demand equals supply) including the labor market, i.e., $L^s = \sum_j L_j$. Therefore, the removal of prolonged periods of sickness or levels of presenteeism are manifested through an increase in the effective-labor supply. In our baseline scenario, we normalize the productivity to the current nocturia prevalence levels by $\overline{E} = 1$. In the *counterfactual* scenarios ($v = 1+, 2+$), we treat (“eliminate”) nocturia for patients that have one or more voids and two or more voids which raises the effective-labor productivity by $E_v = \overline{E} + e_v$. Finally, we compare the baseline with the counterfactual scenarios to obtain the cost of nocturia.

An increase in effective-labor supply is manifested through the removal of prolonged periods of sickness or levels of presenteeism that reduce the effective-labor workforce. In our baseline scenario, we normalize the productivity to the current nocturia prevalence levels by $\overline{E} = 1$. In the *counterfactual* nocturia threshold scenarios ($v = 1+, 2+$), we treat (“eliminate”) nocturia for patients that have one or

more voids and two or more voids which raises the effective-labor productivity by $E_v = \overline{E} + e_v$. Finally, we compare the baseline with the counterfactual scenarios to obtain the productivity cost of nocturia.

The parameter e_v is obtained by

$$e_v = \alpha_v \theta_v \tag{2}$$

with prevalence rate θ_v and work impairment α_v, discussed and estimated econometrically in the paper.

As there is uncertainty related to the parameter inputs, we further test our model assumptions by applying a Monte-Carlo simulation to randomly, independently, generate a range for θ_v and α_v. For each counterfactual scenario, we execute the model 5000 times with randomly generated parameter combinations.

2 Associations between nocturia and work impairment

The table below provides the full regression result including covariates by nocturia definition.

Table 1: Associations between nocturnal voiding and work impairment due to absenteeism or presenteeism (% working time lost)

	(1)	(2)	(3)	(4)
	OLS-3	***FL-3***	***OLS-3***	***FL-3***
**1+ voids**	0.01601	0.01534		
	(0.00183)**	(0.00170)**		
**2+ voids**			0.02186	0.01766
			(0.00352)**	(0.00268)**
**Female**	0.01205	0.01110	0.01256	0.01170
	(0.00213)**	(0.00196)**	(0.00213)**	(0.00196)**
**Age**	-0.00129	-0.00127	-0.00122	-0.00120
	(0.00010)**	(0.00010)**	(0.00010)**	(0.00010)**
**White**	-0.01665	-0.01421	-0.01662	-0.01421
	(0.00548)**	(0.00444)**	(0.00549)**	(0.00444)**
**Asian**	-0.02543	-0.02177	-0.02495	-0.02124
	(0.00851)**	(0.00740)**	(0.00842)**	(0.00732)**
**Black**	0.00287	0.00176	0.00253	0.00134
	(0.00764)	(0.00595)	(0.00764)	(0.00593)
**Irregular hours**	0.01032	0.00799	0.01043	0.00814
	(0.00281)**	(0.00235)**	(0.00282)**	(0.00238)**
**Working hours**	0.00030	0.00034	0.00030	0.00034
	(0.00010)**	(0.00010)**	(0.00010)**	(0.00010)**
**Income (£, 1000s)**	-0.00000	-0.00000	-0.00000	-0.00000
	(0.00000)*	(0.00000)**	(0.00000)	(0.00000)**
**Financial concerns**	0.05733	0.03681	0.05735	0.03695
	(0.00435)**	(0.00270)**	(0.00435)**	(0.00270)**
**No tertiary education**	-0.00920	-0.00872	-0.00906	-0.00858
	(0.00208)**	(0.00199)**	(0.00208)**	(0.00199)**
**Divorced**	0.00969	0.00991	0.00940	0.00972
	(0.00431)*	(0.00385)*	(0.00433)*	(0.00388)*
**Widowed**	0.02414	0.02267	0.02317	0.02189
	(0.01298)	(0.01115)*	(0.01297)	(0.01120)
**Child**	-0.00576	-0.00528	-0.00577	-0.00522
	(0.00168)**	(0.00169)**	(0.00167)**	(0.00168)**
**Engaged**	0.00019	-0.00013	0.00018	-0.00014
	(0.00196)	(0.00192)	(0.00195)	(0.00191)
**Smoker**	0.00430	0.00245	0.00384	0.00193
	(0.00333)	(0.00280)	(0.00331)	(0.00279)
**Excessive alcohol**	-0.00252	-0.00190	-0.00181	-0.00122
	(0.00175)	(0.00171)	(0.00176)	(0.00173)
**Physically inactive**	0.02739	0.02503	0.02713	0.02486
	(0.00190)**	(0.00164)**	(0.00189)**	(0.00163)**

Table 2: (*Continued*)

	(1)	(2)	(3)	(4)
	OLS-3	***FL-3***	***OLS-3***	***FL-3***
**Excessive salt intake**	-0.00404	-0.00239	-0.00411	-0.00254
	(0.00412)	(0.00374)	(0.00411)	(0.00375)
**Obese**	0.01132	0.01016	0.01135	0.01037
	(0.00247)**	(0.00215)**	(0.00248)**	(0.00215)**
**Overweight**	0.00198	0.00250	0.00206	0.00255
	(0.00162)	(0.00158)	(0.00161)	(0.00157)
**Underweight**	0.00015	-0.00041	-0.00058	-0.00115
	(0.00696)	(0.00587)	(0.00694)	(0.00585)
**At risk of mental health problems**	0.21045	0.10733	0.21028	0.10737
	(0.00512)**	(0.00250)**	(0.00509)**	(0.00248)**
**MSK: neck**	0.02006	0.01865	0.02025	0.01882
	(0.00206)**	(0.00182)**	(0.00205)**	(0.00181)**
**MSK: shoulder**	0.01466	0.01313	0.01474	0.01324
	(0.00199)**	(0.00174)**	(0.00199)**	(0.00173)**
**MSK: elbow**	0.00740	0.00345	0.00744	0.00350
	(0.00360)*	(0.00293)	(0.00360)*	(0.00295)
**MSK: wrist/hand**	0.01891	0.01347	0.01891	0.01346
	(0.00231)**	(0.00185)**	(0.00231)**	(0.00186)**
**MSK: upper back**	0.02168	0.01371	0.02173	0.01379
	(0.00240)**	(0.00186)**	(0.00239)**	(0.00185)**
**MSK: lower back**	0.01956	0.01812	0.01987	0.01843
	(0.00151)**	(0.00142)**	(0.00151)**	(0.00143)**
**MSK: hip/thigh**	0.01394	0.00953	0.01391	0.00953
	(0.00238)**	(0.00199)**	(0.00239)**	(0.00201)**
**MSK: knee**	0.00840	0.00694	0.00849	0.00703
	(0.00171)**	(0.00154)**	(0.00172)**	(0.00155)**
**MSK: ankle/foot**	0.01712	0.01293	0.01707	0.01283
	(0.00237)**	(0.00198)**	(0.00238)**	(0.00199)**
**Asthma**	0.01977	0.01601	0.01973	0.01610
	(0.00336)**	(0.00265)**	(0.00335)**	(0.00264)**
**Heart**	0.03937	0.02839	0.03893	0.02821
	(0.00888)**	(0.00644)**	(0.00897)**	(0.00654)**
**Kidney**	0.02163	0.01419	0.02138	0.01401
	(0.01205)	(0.00858)	(0.01205)	(0.00860)
**Cancer**	0.09013	0.06960	0.09025	0.06992
	(0.01603)**	(0.01021)**	(0.01601)**	(0.01028)**
**Diabetes**	0.01508	0.01263	0.01423	0.01180
	(0.00696)*	(0.00554)*	(0.00693)*	(0.00552)*
**Hypertension**	0.02323	0.02023	0.02309	0.02020
	(0.00420)**	(0.00348)**	(0.00418)**	(0.00347)**
**Insomnia**	0.01904	0.01505	0.01922	0.01524
	(0.00087)**	(0.00066)**	(0.00085)**	(0.00065)**
**Short sleep**	0.02308	0.01555	0.02207	0.01471
	(0.00380)**	(0.00265)**	(0.00383)**	(0.00267)**
**Long sleep**	0.02285	0.01874	0.02295	0.01897
	(0.00641)**	(0.00535)**	(0.00642)**	(0.00534)**
**Observations**	52,887	52,887	52,887	52,887

Notes:** p<0.01, * p<0.05. Standard errors in parentheses clustered at the company-level. Dependent variable is percentage of work impairment due to absenteeism and presenteeism (e.g. % of working time lost). Note that estimates need to be multiplied by 100 to receive effects in percentage points. Data sample based on BHW pooled cross-sectional (CS) sample of the years 2017 and 2018. All models in columns 1 to 4 are adjusted for company- and time (week, month and year of given survey response) fixed effects. The models presented in the table mimic the main regression from Table 2 of the main manuscript.

3 References

Aguiar, A., M. Chepeliev, E. L. Corong, R. McDougall, and D. v. d. Mensbrugghe: 2019, 'The GTAP Data Base: Version 10'. *Journal of Global Economic Analysis* **4**(1), 1–27. Number: 1.

Armington, P.: 1969, 'A Theory of Demand for Products Distinguished by Place of Production'. International Monetary Fund (IMF) Staff Papers 16.

Barro, R. J. and X. Sala-i Martin: 2003, *Economic Growth, 2nd Edition*. The MIT Press, 2nd edition.

Hafner, M., E. Yerushalmi, C. Fays, E. Dufresne, and C. Van Stolk: 2020b, 'COVID-19 and the cost of vaccine nationalism'. Publisher: RAND Corporation.

Hafner, M., E. Yerushalmi, M. Stepanek, W. Phillips, J. Pollard, A. Deshpande, M. Whitmore, F. Millard, S. Subel, and C. v. Stolk: 2020d, 'Estimating the global economic benefits of physically active populations over 30 years (2020-2050)'. *British Journal of Sports Medicine* **54**(24), 1482–1487.

Lanz, B. and T. F. Rutherford: 2016, 'GTAPinGAMS: Multiregional and Small Open Economy Models'. *Journal of Global Economic Analysis* **1**(2), 1–77.

Romer, D.: 2000, *Advanced Macroeconomics*. McGraw Hill Higher Education, 2nd revised edition edition.

Rutherford, T. F.: 1999, 'Applied General Equilibrium Modeling with MPSGE as a GAMS Subsystem: An Overview of the Modeling Framework and Syntax'. *Computational Economics* **14**, 1–46.

Yerushalmi, E., P. Hunt, S. Hoorens, C. Sauboin, and R. Smith: 2019, 'Exploring the Use of a General Equilibrium Method to Assess the Value of a Malaria Vaccine: An Application to Ghana'. *Medical Decision Making (MDM) Policy & Practice* **4**(2).
